# Predictive Score for Carbapenem-Resistant Gram-Negative Bacilli Sepsis: Single-Center Prospective Cohort Study

**DOI:** 10.3390/antibiotics12010021

**Published:** 2022-12-23

**Authors:** Marisa Zenaide Ribeiro Gomes, Douglas Quintanilha Braga, Debora Otero Britto Passos Pinheiro, Renata Cristina Amorim Silveira Verduc, Letícia Vellozo dos Reis, Elisangela Martins de Lima, Newton Dias Lourenço, Patrícia Aquen Cid, Debora Souza Beck, Luiz Henrique Zanata Pinheiro, João Pedro Silva Tonhá, Luiza Silva de Sousa, Mayra Lopes Secundo Dias, Amanda Aparecida da Silva Machado, Murillo Marçal Castro, Vitoria Pinson Ruggi Dutra, Luciana Sênos de Mello, Maxuel Cassiano da Silva, Thaisa Medeiros Tozo, Yann Rodrigues Mathuiy, Lucas Lameirão Pinto de Abreu Rosas, Paulo Cesar Mendes Barros, Jeane Oliveira da Silva, Priscila Pinho da Silva, Carolina Souza Bandeira, Scyla Maria de Sant′Anna Reis Di Chiara Salgado, Marcio Zenaide de Oliveira Alves, Roberto Queiroz Santos, José Aurélio Marques, Caio Augusto Santos Rodrigues, Saint Clair dos Santos Gomes Junior

**Affiliations:** 1 Laboratório de Genética Molecular de Microrganismos, Instituto Oswaldo Cruz, Oswaldo Cruz Foundation (IOC/FIOCRUZ), Rio de Janeiro 21040-900, Brazil; 2 Laboratório de Pesquisa em Infecção Hospitalar, Instituto Oswaldo Cruz, Oswaldo Cruz Foundation (IOC/FIOCRUZ), Rio de Janeiro 21040-900, Brazil; 3 Hospital Federal dos Servidores do Estado (HFSE), Ministry of Health, Rio de Janeiro 20221-903, Brazil; 4 Hospital Infection Control Committee, Hospital Universitário Pedro Ernesto, Rio de Janeiro State University, Rio de Janeiro 20551-030, Brazil; 5 Instituto Fernandes Figueira, Oswaldo Cruz Foundation, Rio de Janeiro 22250-020, Brazil

**Keywords:** sepsis, Gram-negative bacteria, antibiotic resistance, risk scores, cohort study, ROC curve, intensive care unit

## Abstract

A clinical–epidemiological score to predict CR-GNB sepsis to guide empirical antimicrobial therapy (EAT), using local data, persists as an unmet need. On the basis of a case–case–control design in a prospective cohort study, the predictive factors for CR-GNB sepsis were previously determined as prior infection, use of mechanical ventilation and carbapenem, and length of hospital stay. In this study, each factor was scored according to the logistic regression coefficients, and the ROC curve analysis determined its accuracy in predicting CR-GNB sepsis in the entire cohort. Among the total of 629 admissions followed by 7797 patient-days, 329 single or recurrent episodes of SIRS/sepsis were enrolled, from August 2015 to March 2017. At least one species of CR-GNB was identified as the etiology in 108 (33%) episodes, and 221 were classified as the control group. The cutoff point of ≥3 (maximum of 4) had the best sensitivity/specificity, while ≤1 showed excellent sensitivity to exclude CR-GNB sepsis. The area under the curve was 0.80 (95% CI: 0.76–0.85) and the number needed to treat was 2.0. The score may improve CR-GNB coverage and spare polymyxins with 22% (95% CI: 17–28%) adequacy rate change. The score has a good ability to predict CR-GNB sepsis and to guide EAT in the future.

## 1. Introduction

Early recognition and proper empirical antimicrobial treatment (EAT) of sepsis to cover carbapenem-resistant Gram-negative bacilli (CR-GNB) are fundamental in changing the current scenario of high mortality rates [1,2]. These are especially important because therapeutic options for the treatment of CR-GNB infection are still limited [3,4]. CR-GNB, such as *Acinetobacter baumannii*, *Pseudomonas aeruginosa*, and *Klebsiella pneumoniae*, are increasingly being reported worldwide, although with some regional and hospital variations [2,3,5]. In the hospital studied, these agents have become the main cause of sepsis in the intensive care units (ICUs) for adults [5], followed by Gram-positive bacteria and fungi, in monomicrobial or polymicrobial infections [2,3,5].

Geographic and temporal variations, as well as differences in antimicrobial response, highlight the clinical–epidemiological importance of the carbapenem resistance mechanisms involved [6]. Phenotypic resistance to carbapenems is usually caused by Amber class A, B, and D β-lactamases. Additionally, extended-spectrum β-lactamases and AmpC cephalosporinases, when combined with porin mutation or the overexpression of efflux pumps, can lead to carbapenem resistance [7]. Nonenzymatic carbapenem resistance includes loss of expression or mutations of porin-encoding genes, and the overexpression of genes encoding efflux pumps, particularly in *P. aeruginosa* [7,8]. Rarely, mutations or other modifications that alter the level of production or affinity of penicillin-binding proteins may also be responsible for resistance to carbapenems [7,9]. Intrinsic resistance to carbapenems is present in some species, such as *Stenotrophomonas maltophilia* [7]. In addition, the concomitance of resistance to other classes of antimicrobials is common among CR-GNB isolates [7,9]. 

EAT should be started within the first hour of the presumed diagnosis of sepsis, at a time when the clinical–epidemiological characteristics remain as the only determinants of patient risk [5]. Risk score studies based on clinical–epidemiological parameters with the aim of assisting physicians in choosing the most appropriate EAT for sepsis remain scarce or nonexistent [10,11,12]. One study was conducted in a tertiary-level hospital in the Netherlands, where resistance rates were low and EAT was based on the combination of second- or third-generation cephalosporin associated with aminoglycoside; thus, this did not reflect our reality. Through risk-based strategies, the authors were able to estimate the possibility of reducing carbapenem usage by 83%, associated with a treatment adequacy rate of 96% [11]. In another study, the accuracy of a risk score was evaluated in predicting the first episode of imipenem-resistant GNB bacteremia, predominantly of non-fermenters, among critically ill patients in a tertiary hospital in Taiwan, in 2016 [10]. The obtained predictor model showed good discrimination with an area under the receiver operating characteristic (ROC) curve (AUC) of 0.75 (95% CI: 0.70–0.80) through internal validation.

The study design and the concordance of our data with the literature on the risk factors for CR-GNB presented in our previous publication support the utility in developing a predictive score to stratify patients according to the risk of CR-GNB sepsis at the first encounter [5]. In this study, our overall aim was to develop and estimate the performance of a predictive score for sepsis caused by CR-GNB in the entire cohort. Our hypothesis was that a score based on clinical–epidemiological predictors, developed locally, would have sufficient accuracy to identify patients at high risk of CR-GNB sepsis, among adult patients receiving intensive care. In addition, the adequacy of carbapenem and polymyxin therapy, as well as EAT, was investigated, with the aim of determining the proportion of patients in which the score system would effectively improve the EAT.

## 2. Materials and Methods

### 2.1. Patients, Setting, Study Design, and Methodology 

This study was an extension of a case–case–control study that determined risk factors for sepsis caused by CR-GNB [5], based on a prospective cohort study of patients with systemic inflammatory response syndrome (SIRS), sepsis-2 [13], or adapted sepsis-3 [5] criteria (Supplementary Table S1), in which blood cultures were collected and antimicrobial therapy was initiated, for two or more days, in an adult clinical surgical ICU at a 450-bed tertiary public federal general hospital in Rio de Janeiro, Brazil, from August 2015 to March 2017. The study was approved by the institutional ethics committee and adhered to the Declaration of Helsinki and its later amendments. 

**Figure 1:**
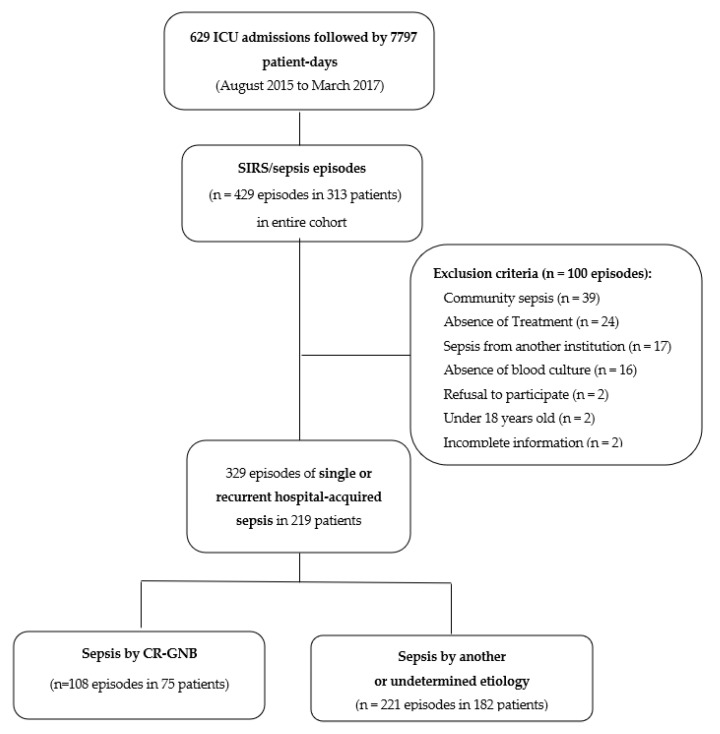
Flowchart of patients included in the study. CR-GNB, carbapenem-resistant Gram-negative bacilli.

We investigated all single or recurrent episodes of sepsis following all blood cultures and other clinical and surveillance cultures during ICU stay, as well as the antimicrobial therapy instituted previously and in the 30 day follow-up period after the end of sepsis treatment, throughout the cohort study. The cohort study and the case–case–control methodology were designed to determine risk factors for CR-GNB sepsis, as previously described [5]. In this study, all episodes of hospital-acquired sepsis identified during the follow-up period were recorded, including all recurrent episodes of sepsis from the same patient. We only excluded episodes of sepsis acquired in the community or those associated with another healthcare institution, as well as incidences in patients younger than 18 years old or those who refused to sign the consent form (Figure 1). We did not exclude polymicrobial infections. Therefore, all episodes with at least one species of CR-GNB as the etiology of sepsis were classified as Group 1 (CR-GNB sepsis group); Group 2 comprised all other episodes.

The case–case–control methodology, designed to define risk factors for CR-GNB sepsis, was described previously [5]. The variables and definitions, the information sources, the microbiological methods, the forms used, and the checklist of STROBE recommendations [14] of the cohort study have also already been published [5]. The sepsis-3 criteria [15] were used in an adapted manner and applied retrospectively in a random sampling of the cases included in the cohort [5], with regard to increases in two or more points of the Sequential Sepsis-Related Organ Failure Assessment (SOFA), while also considering the qSOFA when the patient no longer required mechanical ventilation or sedation. Recurrent sepsis was defined as a new episode of sepsis developing after the resolution of clinical and laboratory parameters of sepsis or the recrudescence of sepsis with evidence of new etiology by cultures during the ICU stay [5]. 

The following variables were also investigated at ICU admission: demographic data, origin and reason for hospitalization and clinical severity scores, such as the Charlson comorbidity index, Simplified Acute Physiology Score (SAPS 3), and SOFA. The following variables were investigated prior to the blood culture assessment upon entry to the study in each episode of sepsis: comorbidities, length of previous hospitalization in the hospital and ICU, invasive devices (central vascular catheter, mechanical ventilation, and indwelling bladder catheter), hemodialysis, enteral and parenteral nutrition, the SOFA score, and C-reactive protein levels. In addition, we investigated the etiology and type of infection that motivated sepsis, using the strict criteria developed by Klein Klouwenberg et al., (2013) [16], adapted to include Centers for Disease Control and Prevention definitions of healthcare-associated infections [17], the presence of concurrent infection, and the outcomes for discharge or death in the ICU and hospital. 

To avoid potential bias, all data collected were standardized and monitored throughout the study. Post hoc analysis was performed to review all clinical, radiological, and microbiological data, including the evidence of sepsis-2 [13] and -3, and the etiology and source of infection. We also classified the plausibility of infectious source as being definitive, probable, possible, or undetermined [16]. 

In this cohort study, antimicrobial therapy was investigated whenever used up to the 3 days prior to the initial blood culture collection, as was empirical antimicrobial therapy comprising the period between blood culture collection and the final microbiological results that determined or not the etiology of sepsis. All the antimicrobial drugs maintained or initiated after initial blood culture collection that had no other reason to be used were considered to be for the treatment of the investigated sepsis episode. Adequate empirical treatment was defined as the use of any antimicrobial regimen with at least one drug susceptible to cover the etiology of sepsis. 

### 2.2. Database, General Statistical Analyses, and Risk Score Development 

We collected and managed study data using REDCap electronic data capture tools hosted at Instituto Oswaldo Cruz, Fundação Oswaldo Cruz (IOC/FIOCRUZ) [18]. All statistical analyses were performed using SPSS® statistics v22.0 software. Categorical variables were compared using the exact chi-square or Fisher tests; continuous variables were compared using the Mann–Whitney–Wilcoxon test [19]. 

Predictive factors for CR-GNB sepsis have been defined previously: (1) infection prior to the sepsis episode (OR = 4.28; 95% CI: 1.77–10.35; *p* = 0.001); (2) previous use of mechanical ventilation (OR = 4.21; 95% CI: 1.17–15.18; *p* = 0.028); (3) previous use of carbapenem (OR = 3.42; 95% CI: 1.37–8.52; *p* = 0.008); (4) length of hospital stay (OR 1.03; 95% CI: 1.01–1.05; *p* = 0.007), which were scored according to the logistic regression coefficients (Supplementary Table S2). ROC curve analysis determined the accuracy of the score in predicting sepsis by CR-GNB in the entire cohort by assessing the area under the curve (AUC) [20]. Therefore, the risk factors independently associated with CR-GNB sepsis, identified on the basis of this cohort study and using the case–case–control design [5], were subsequently scored in order to develop a score system. The score was applied in all episodes included in the cohort, regardless of whether the etiology was identified, in order to better estimate its ability to predict CR-GNB sepsis throughout the population studied as real life.

The case–case–control methodology is recommended for the investigation of antimicrobials as factors associated with bacterial resistance profiles [5,21]. Thus, points were attributed to each variable determined, in our previous publication [5], as a predictor of CR-GNB sepsis by multivariate analysis, using the logistic regression coefficients (Supplementary Table S2), as recommended [22]. The discriminatory capacity of the clinical risk score was calculated by applying it in all sepsis episodes in this study, regardless of whether it was recurrent or single, because each episode was treated as an individualized event; all predictive factors were investigated during the hospitalization period prior to the initial blood culture. In addition to sensitivity, specificity, negative predictive value (NPV), positive predictive value (PPV), and accuracy, we report the Youden index, calculated according to standard methods [10,20]. These indicators were used at various cutoff points to determine the best cutoff to discriminate high and low risk of CR-GNB as the etiology of sepsis. The proportion of episodes scoring 0, 1, 2, 3, and 4 points in each study group was also calculated, as well as in the set of sepsis caused by CS-GNB (carbapenem-susceptible Gram-negative bacilli), non-GNB sepsis, and undefined etiology, in order to better understand the behavior of the score in all episodes occurring in the cohort.

We analyzed the number needed to treat (NNT) to avoid empiric therapy divergence in one patient [23]. The uses of empiric carbapenems, empiric polymyxins, and adequate EAT were also analyzed in both study groups and according to the scoring system, so as to calculate the proportion of patients in which the score would be useful to increase or avoid CR-GNB coverage. 

The adequation rate of the score was calculated considering the number of episodes in which it would be necessary to receive empiric polymyxin therapy to cover CR-GNB (true positive), plus the number of episodes in which empiric polymyxin therapy could be avoided (true negative), divided by the total number of episodes. Both sides of the curve and a significance level of 5% were considered in all tests. 

## 3. Results

### 3.1. Patients and Episodes of Sepsis

Of a total of 629 ICU admissions followed by 7797 patient-days, we identified 429 single or recurrent episodes of SIRS/sepsis in 313 patients after having completely monitored the entire cohort. We enrolled 329 episodes of hospital-acquired sepsis identified in 219 patients. Approximately 33% (108/329) of the episodes had at least one species of CR-GNB as the cause of sepsis, which were classified as Group 1 (108 episodes in 75 patients). Group 2 comprised 221 sepsis episodes in 182 patients, caused by CS-GNB (26%, 57/221), non-GNB agents (23%, 50/221), or an undetermined etiology (56%, 124/221) (Figure 1). Recurrent episodes of hospital-acquired sepsis were identified in 30% (65/219) of patients during ICU stay; 18% (39/219) were allocated in both study groups. We identified a median of one (range of 1–10) episode in the study population and a ratio of 1.4 episodes per patient in CR-GNB group versus 1.2:1 among the control group (*p* = 0.15). Table 1 and Table 2 present the profiles of patients and episodes in each group of the study, respectively.

The total SOFA score exhibited median values of seven (mean 7.1; SD 3.5; range 1–17 among Group 1 and mean 6.6; SD: 3.11; range 0–18 in Group 2) and eight points (mean 7.7; SD 4.2; range 0–20 among group 1 and mean 7.4; SD 4.3; range 0–18 in Group 2) in both groups, at ICU admission and at initial blood culture (*p* = 0.89), respectively, showing no difference in the degree of dysfunction or previous organic failure from the univariate analysis (Table 2). Nosocomial diarrhea (*p* < 0.001), infection/colonization by any CR-GNB prior to sepsis episode (*p* < 0.001), and previous infection (mostly hospital-acquired bacterial infection or sepsis) (*p* < 0.001) were more frequent comorbidities in CR-GNB sepsis than non-CR-GNB sepsis. The use of any mentioned invasive devices was significantly higher among episodes of CR-GNB sepsis than in the comparison group, such as the use of enteral nutrition (*p* < 0.001) (Table 2). All the CR-GNB sepsis episodes were treated with antimicrobials prior to the collection of blood culture, as compared with 82% in those with another or indeterminate etiology (*p* < 0.001), with significant differences exhibited in carbapenem (88% versus 42%, *p* < 0.001) and polymyxin (57% versus 23%, *p* < 0.001) usage. The median lengths of hospital and ICU stays prior to the incidence of sepsis were significantly higher in the episodes of CR-GNB sepsis than in episodes of sepsis due to another or undetermined etiology (*p* < 0.001). The 30 day mortality rate (66%, 95% CI: 47–89% versus 44%, 95% CI: 34–55%, *p* = 0.004) and hospital mortality rate (70%, 95% CI: 51–94% versus 55%, 95% CI: 44–68%, *p* = 0.038) were significantly higher in patients with CR-GNB sepsis than in comparators, considering the last or unique episode of sepsis identified in each patient. 

### 3.2. Etiology and Infectious Focus of Sepsis 

The distribution of infectious foci and its plausibility (possible, probable, or definitive) by groups of study are shown in Supplementary Figures S1 and S2. Among the sites of infection, ventilator-associated pneumonia (VAP) was more prevalent in the CR-GNB sepsis group than in the comparison group (47% and 10%, respectively, *p* < 0.001), whereas non-ventilator-associated hospital-acquired pneumonia was more common in Group 2 than in the CR-GNB group (23% and 13%, respectively, *p* = 0.04) (Table 2). 

Among all episodes included in this study, we identified 162 isolates causing monomicrobial and polymicrobial (43%, 46/108) sepsis in 108 episodes in 75 patients (Supplementary Figure S3). In total, 124 CR-GNB isolates were identified; at least one carbapenem-resistant isolate was identified in each episode in the CR-GNB group. CR-non-fermenting bacilli were the most frequent occurring in 69% (75/108) of the cases (43%, 32/75 polymicrobial). *Acinetobacter baumannii* (43%; 46/108) accounted for one-third of the cases. *Pseudomonas aeruginosa*, *Stenotrophomonas maltophilia*, and *Burkolderia cepacea* represented 26% (28/108), 12% (13/108), and 1% (1/108), respectively. Among the species of the family *Enterobacteriaceae* resistant to any carbapenems (CREs), responsible for 31% (34/108) of the episodes (65%; 22/34 polymicrobial), *Klebsiella pneumoniae* was identified in 19% (20/108), followed by *Providencia stuartii* (6%; 6/108), *Proteus mirabillis* (5%; 5/108), *Enterobacter cloacae* (2%; 2/108), *Serratia marcescens* (2%; 2/108), and *Klebsiella aerogenes* (1%; 1/108). 

Among episodes of sepsis classified as another etiology or non-CR-GNB (Supplementary Figure S3, gray bars), 119 isolates were identified in 97 episodes, causing 20 (21%, 20/97) cases of polymicrobial sepsis. CS-GNB accounted for approximately half of the strains (51%, 61/119) and 59% (57/97) of episodes (23%, 13/57 polymicrobial). Species of the *Enterobacteriaceae* family were the most frequent (62%; 38/61 in 37 episodes), and non-fermenting bacilli represented 39% (24/61 in 23 episodes). Gram-positive cocci accounted for 36% (43/119) of isolates in 36 episodes (22%, 8/36 polymicrobial), with the predominance of *Staphylococcus* species (60%, 26/43 in 22 episodes): *S. aureus* (11%; 13/119 in 13 episodes) and coagulase-negative staphylococci (11%; 13/119 in 10 episodes). *Streptococcus* and *Enterococcus* species were identified in 8% (9/119 in eight episodes) and 6% (7/119 in seven episodes) of agents, respectively. *Candida albicans*, non-albicans *Candida* spp., and yeasts were identified in 8% (9/119) of isolates in nine episodes, whereas *Mycobacteria tuberculosis* (diagnosed during hospitalization period or health-care associated) and other agents (*Moraxella* sp., *Haemophylus* sp., and anaerobic Gram-positive bacilli) were responsible for three episodes each. 

### 3.3. Performance of the Clinical–Epidemiological Score to Guide EAT

**Figure 2:**
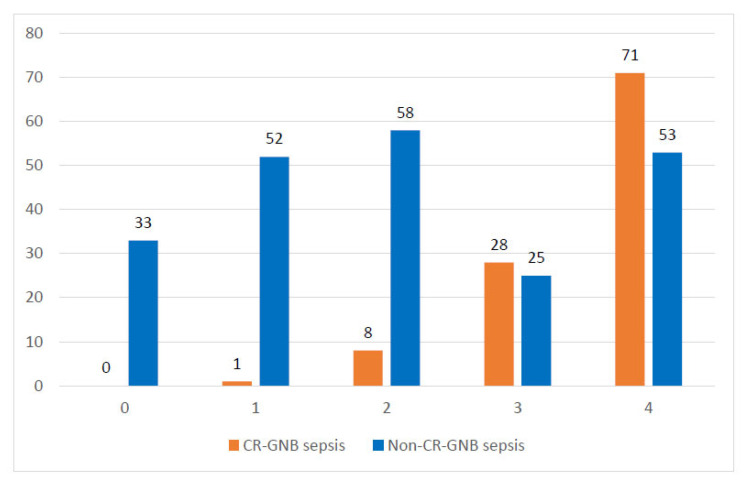
Distribution of groups of sepsis episodes according to the score obtained from the case–case–control design study already published [5]. Score for CR-GNB sepsis: infection prior to the sepsis episode, one point; previous use of mechanical ventilation, one point; previous use of carbapenem, one point; length of hospital stay greater than or equal to 19 days, one point. Each score factor present contributed to one point for the final score in each sepsis episode in the entire cohort.

**Figure 3:**
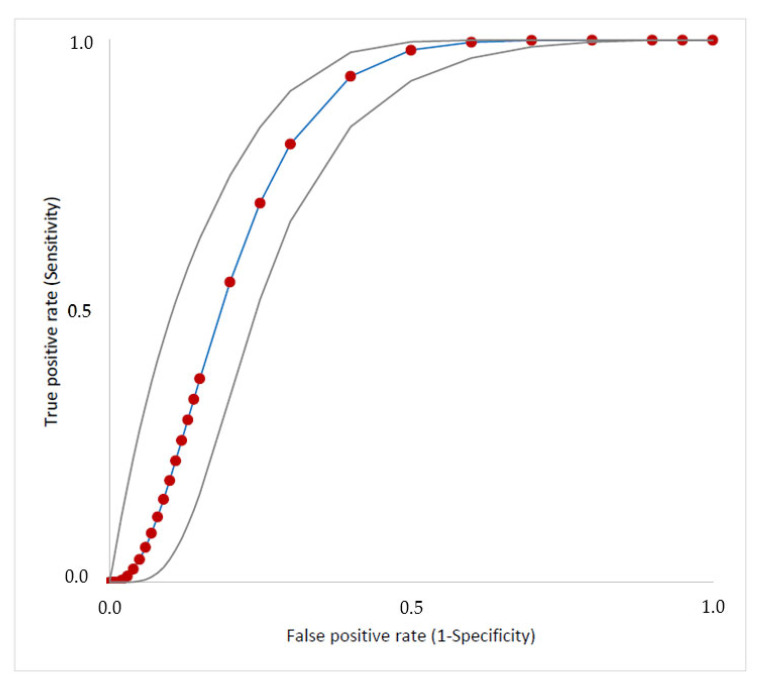
Receiver operating characteristic curve for the prediction of CR-GNB in sepsis as a function of the four-point score defined in Supplementary Table S2. Area under the curve of 0.80 (95% CI: 0.76–0.85, *p* < 0.001). Upper and lower boundaries are shown in gray.

The sensitivity, specificity, numbers of false negatives and false positives, negative predictive value (NPV), positive predictive value (PPV), and accuracy of clinical score using different cutoff points are presented in Table 3. Sepsis episode distributions in both groups according to the score obtained are shown in Figure 2. The AUC value of the ROC curve was 0.80 (95% CI 0.76– 0.85, *p <* 0.001) (Figure 3).

We observed that when a cutoff point ≥ 3 was used, we found a high sensitivity (92%, 95% CI, 85–96%), with a significant reduction in false negatives, when compared with a score of four, and the best accuracy (74%, 95% CI 69–78%) in discriminating episodes with a higher risk for CR-GNB sepsis (Table 3). In contrast, a score ≤ 1 demonstrated high sensitivity (99%; 95% CI: 95–100%) to exclude those at risk of CR-GNB sepsis. The performance of a clinical–epidemiological score ≥ 3 to predict CR-GNB sepsis, in accordance with the etiology of sepsis, score distribution, and prior use of polymyxins, is shown in Supplementary Table S3. The NNT using this cutoff for an additional episode to benefit CR-GNB coverage was 2.0 (95% CI: 1.5–2.1, *p* < 0.0001). 

The proportions of carbapenem and polymyxin usage before (3 days preceding) and after (empirical therapy) initial blood culture in CR-GNB and non-CR-GNB sepsis groups (Supplementary Figure S4), in accordance with the etiology of sepsis and the score distribution, are displayed in Supplementary Figures S5 and S6. Despite a previously high intake of carbapenems and polymyxins as EAT in the study population, with significant differences between the study groups (Supplementary Figure S4) and by etiology (Supplementary Figure S5), 21% (23/108; 95% CI: 14–30%) and 25% (27/108; 95% CI: 17–34%) of patients with CR-GNB sepsis did not receive polymyxins or adequate EAT, respectively (Supplementary Figures S4 and S7). Moreover, in 94% (84/85) of the episodes that registered scores of 0 (n = 33) and 1 (n = 52), representing 26% (84/329, 95% CI: 21–31%) of study episodes (Supplementary Table S4), the use of polymyxins could perhaps be spared by applying the score. The adequacy rates of CR-GNB coverage in clinical practice (pre-test, 42%, 85/205; post-test, 131/205, 64%) showed a 22% (95% CI: 17– 28%) variation in favor of the use of the score (Supplementary Table S5). 

## 4. Discussion 

The prevalence of CR-GNB sepsis is increasing globally, and the rational use of antibiotics is one of the pillars to contain the agents and extend the shelf-life of antibiotics [24,25,26]. Physicians around the world, particularly those in ICUs, need to use effective antimicrobial treatments as quickly as possible to prevent disability and death in patients without indiscriminately prescribing broad-spectrum antibiotics [27]. When to escalate sepsis therapy to cover these microorganisms, as well as how to preserve last-resort antibiotics, particularly newly developed drugs, are frequent impasses. The solution to this problem seems simple, but it is not to prescribe the antibiotic with just the right spectrum. Thus, a clinical score to help decide whether or not to expect antimicrobial resistance at the bedside when deciding EAT, especially with easy-to-use components, is of great importance.

From an antimicrobial stewardship standpoint, it is recommended that EAT be adjusted to the local data [11], according to the local epidemiology of patients and pathogens. The developed score is a simple tool, because the predictor variables can be easily obtained during initial assessments, at a time when the clinical–epidemiological characteristics remain the only determinants of a patient´s risk. The score aims to guide EAT until a timely antimicrobial de-escalation based on microbiological identification, susceptibility testing, and clinical response is implemented. These are essential strategies to improve the prognoses of septic patients, preserving the effectiveness of existing antimicrobials and preventing the emergence of resistance. In our study, the score represented a valuable option to effectively improve polymyxin coverage in 21% (14–30%) of CR-GNB sepsis cases and may be useful to withdraw unnecessary coverage in 26% (95% CI: 21–31%) of the studied population. 

To the best of the authors’ knowledge, this is the first study to develop a suitable predictive score to guide EAT for CR-GNB sepsis. Although bacteremia has been widely used as a proxy for sepsis, in our study, bacteremia did not represent all types of infections and was identified in a smaller proportion of cases, mainly catheter related bloodstream infections [5]. Bacteremia has been identified in fewer than 30% of septic cases in ICUs [5,28,29], and blood culture is relatively insensitive for the recovery of agents in VAP [30,31], the predominant infectious source of ICU sepsis [2,29]. In addition, a diversity of GNB species could be the cause of sepsis in monomicrobial and polymicrobial infections as demonstrated, making the score for specific species not useful for targeting therapy. Considering incident cases, all single and recurrent episodes in the cohort, and the variety of infections commonly identified in hospital-acquired sepsis, the estimates are likely to be more representative of what happens in real life. In addition, including episodes with unknown etiology improves our understanding of the complex epidemiological picture, aiming to understand the balance between damages and benefits of novel tool use. 

Successful longitudinal follow-up of this cohort was essential to achieve the study proposal. Prior infection, mechanical ventilation, carbapenem usage, and hospitalization longer than or equal to 19 days were associated with a higher risk of sepsis due to CR-GNB [5], in agreement with the literature concerning the risk factors for the acquisition of or infection by CR-GNB [32,33,34,35]. 

Using data from a prospective cohort, we developed a predictive score that allowed us to accurately identify patients at high or low risk of CR-GNB sepsis. The score performance was considered to be satisfactorily estimated when applied to the entire cohort. The best combination of sensitivity (92%, 95% CI: 85–96%) and specificity (65%. 95% CI: 58–71%) was reached when we used three or more points as cutoffs. The best specificity was achieved using four as the cutoff (76%, 95% CI: 70–81%). A score of less than or equal to one showed optimal sensitivity (99%; 95% CI: 95–100%) for the exclusion of CR-GNB sepsis cases. Those exhibiting scores of two had at least 8% (8/96) false negative results, which is high considering the severity of clinical cases. Notably, higher scores (three and four) had 25% and 29% occurrences of inappropriate EAT, respectively, and these patients would benefit with a broader-spectrum EAT if the score were to be applied prospectively. Patients with lower scores (zero and one) used polymyxins unnecessarily, while exerting pressure on antimicrobial resistance and incrementing cost. Sepsis by non-GNB (including Gram-positive bacteria) also had 35% inadequate EAT, although the score would not solve all the issues, because it is designed for CR-GNB.

Additionally, the inhibition of CR-GNB growth in cultures due to polymyxin use could explain false positive or discordant results, because the drug was administered in 40% of these cases within the 3 days preceding the collection of cultures. Therefore, some of the episodes in the control group would have been in the case group if the agent was identified by microbiological tests. Thus, considering the high incidence of CR-GNB sepsis and high consumption of carbapenem and polymyxins, we expect that the score has the potential to benefit a larger number of patients who are misclassified as controls. Consequently, an NNT of two to avoid mismatch in a single case is probably an overestimation. 

Taking everything in account, our findings indicate that episodes with scores of three or four should be treated with the best drugs available to cover CR-GNB sepsis, knowing that we may need to treat two patients to adequately cover one. For those exhibiting scores of zero and one, the drugs can be preserved. However, episodes presenting a score of two should be handled on a case-by-case basis. Considering that 77% (144/186) of the episodes with scores of three or four had an etiology later determined by conventional cultures, and a higher proportion would be achieved if molecular tests were incorporated into the diagnosis of sepsis [36], broad-spectrum EAT initially guided by the new score with subsequent adjustment of therapy, according to the microbiological results, contributes to its incorporation into clinical practice. Compared with standard therapy (clinical practice), we estimated an increase in the adequacy rate, considering the use of empiric polymyxins as a proxy for adequate CR-GNB cover, in 22% (95% CI: 17–28%) of study episodes using the CR-GNB sepsis score. A more in-depth analysis, however, of our results is still under evaluation to indicate the best strategy for the applicability of the score in future studies. 

In fact, in this scenario of a high prevalence of CR-GNB sepsis, as we demonstrated, high intakes of both carbapenems and polymyxins are expected, because these drugs remain the most widely used drugs in double or triple combinations for the treatment of CR-GNB sepsis, especially in hospitals with restricted access to novel therapeutic options. EAT with different combinations of broad-spectrum antibiotics, generally using the old drugs polymyxins, tigecycline, amikacin, and/or meropenem, is the standard treatment regimen for hospital-acquired septic patients in public Brazilian ICUs, despite the reports of increased resistance [37]. The emergence of resistance to newer agents, such as ceftazidime–avibactam, is critical and further intensifies this problem [38]; therefore, using a bedside tool to preserve the administration of expensive wide-spectrum antibiotics in critically ill patients with a low score (≤1), limiting the use to those with high scores (≥3), indicates the importance of the developed tool and its potential.

The main limitations include the single-center nature of the study, which warrants caution for any generalization of our findings. Internal validation is required before implementation, and external, multicentric validations in hospitals with a similar size, profile, and prevalence rates of CR-GNB sepsis are important before extrapolating these results beyond our cohort. The adaptation of management strategies to the local epidemiological data is a general recommendation for hospital infection, which indicates locally cyclic evaluations. The performance of microbiological methods and the use of antimicrobials, which may have inhibited microbial growth in cultures, could also have influenced our estimates. Nonfermenting CR-GNB were more frequent than *Enterobacterales* CR-GNB in this cohort, whereas carbapenemase production was previously estimated in 76% of CR-GNB isolates [5]. These factors should be taken into consideration depending on the sepsis microbiology profiles across institutions, as well as the types of microbiological monitoring, once VAP is likely less represented by bacteremia [30,31]. 

## 5. Conclusions

Developing a predictive score for empirical sepsis therapy based on local microbiological and clinical data, and estimating NNT CR-GNB sepsis to avoid inadequate EAT and overtreatment in a patient are feasible approaches. Such a score was proposed in this study, and its overall adequacy rate in not only indicating the correct treatment of CR-GNB, but also in avoiding the overtreatment of such pathogens, sparing antibiotics such as polymyxins, is promising. However, defining strategies for the EAT of sepsis is very ambitious, considering limitations in the drug arsenal, costs, drug–drug interactions, and adverse events, as well as the clinical severity of cases. This also comprises moral principles, because decisions of when and how to use EAT affects current patients, as well as future generations. The emergence of resistance to novel drugs is a serious issue; therefore, more clinical studies are needed to determine the ideal approach for EAT, especially in the high-incidence setting of CR-GNB sepsis (33%), as well as for patients with severe sepsis and septic shock, such as the majority of our cases. 

## Figures and Tables

**Table 1:** Clinical and demographic characteristics of patients with episodes of CR-GNB sepsis and sepsis due to another or undetermined etiology (non-CR-GNB sepsis).

	CR-GNB Sepsis (n = 75)	Non-CR-GNB Sepsis (n = 182)
**Demographic data**		
Age in years, median (range)	62 (23–91)	63 (19–92)
Male, n (%)	39 (52)	72 (40)
Prior ICU admission, n (%)	17 (23)	30 (16)
**ICU admission reason, n (%)**		
Infection	53 (71)	94 (52)
Sepsis	41 (55)	68 (37)
Respiratory failure	32 (43)	58 (32)
Septic shock	29 (39)	59 (32)
Respiratory disease	20 (27)	48 (26)
Surgery	17 (23)	60 (33)
Elective surgery	11 (15)	35 (19)
Urgent surgery	6 (8)	25 (14)
Cardiovascular disease	12 (16)	23 (13)
Renal and urinary tract disease	10 (13)	23 (13)
Gastrointestinal or intrabdominal disease	9 (12)	20 (11)
Neurological disease	3 (4)	19 (10)
**Charlson comorbidity index ^a^**, median (range)	2 (0–19)	2 (0–11)
**Total SOFA score ^a^**, median (range)	7 (1–17)	7 (0–18)
SOFA parameters ≥2 points, % (n/valid))		
Cardiovascular	56 (42/75)	51 (92/181)
Respiratory	45 (61/74)	50 (89/178)
Renal	38 (28/74)	39 (70/180)
Neurological	34 (21/61)	28 (42/152)
Liver	15 (10/68)	16 (24/149)
Coagulation	13 (10/75)	16 (28/178)
**SAPS 3 score ^a^**, median (range)	64 (24-103)	62 (24–114)
**Mortality rate ^b^, % (n)**		
**30 day mortality**	66 (42/64)	44 (68/154)
**Hospital mortality**	70 (45/64)	55 (*85/154)*

^a^ At admission; ^b^ considering the last or unique episode of sepsis detected in each patient. Abbreviations: CR-GNB, carbapenem-resistant Gram-negative bacilli; SOFA, Sequential Organ Failure Assessment; SAPS 3, Simplified Acute Physiology Score III.

**Table 2:** Clinical features of sepsis episodes caused by CR-GNB and by another or undetermined etiology (non-CR-GNB sepsis).

Clinical Factors	CR-GNB Sepsis (n = 108)	Non-CR-GNB Sepsis (n = 221)	*p*-Value ^i^
**Comorbidity ^a^**, n (%)			
Previous infection	101 (94)	117 (53)	<0.001 *
Renal failure	57 (53)	87 (39)	0.02 *
CR-GNB Infection/Colonization	52 (48)	43 (20)	<0.001 *
Previous surgeries	50 (46)	122 (55)	0.13
Diabetes mellitus	49 (45)	87 (39)	0.16
Hemodialysis	46 (43)	57 (26)	0.002 *
Nosocomial diarrhea ^b^	31 (29)	21 (10)	<0.001 *
Neoplasia	28 (26)	79 (36)	0.74
Pulmonary disease	27 (25)	44 (20)	0.29
Gastrointestinal disease	27 (25)	51 (23)	0.70
Immunosuppressive condition ^c^	26 (24)	34 (15)	0.06
Genitourinary disease	9 (8)	21 (10)	0.73
Neutropenia ^d^	6 (6)	6 (3)	0.20
Chronic liver disease	5 (5)	25 (11)	0.06
AIDS or chronic infectious disease	4 (4)	11 (5)	0.60
Pregnancy	4 (4)	5 (2)	0.45
**Invasive devices ^a^**, n (%)			
Central vascular catheter	108 (100)	192 (87)	<0.001 *
Mechanical ventilation	104 (96)	149 (67)	<0.001 *
Indwelling urinary catheter	97 (90)	179 (81)	0.04 *
**Nutrition**, n (%)			
Enteral nutrition	93 (87)	147 (67)	<0.001 *
Parenteral nutrition	69 (64)	116 (53)	0.05
**Length of hospital stay ^e^**, median (range)	54 (4–292)	27 (0–340)	<0.001 *
**Length of ICUl stay ^e^**, median (range)	33 (0–291)	12 (0–339)	**<0.001**
**Previous antimicrobial use**, n (%)	108 (100)	180 (81)	<0.001 *
**Previous carbapenem use**, n (%)	95 (88)	93 (42)	<0.001 *
**Previous polymyxin use**, n (%)	62 (57)	51 (23)	<0.001 *
**SIRS ^f^**, n (%)	106 (98)	217 (99)	0.34
**Total SOFA score ^g^**, n (%)	8 (0-20)	8 (0–18)	0.60
SOFA parameters ≥ 2 points, % (n/valid))			
Cardiovascular	62 (66/107)	60 (130/217)	0.76
Respiratory	53 (56/106)	53 (114/215)	0,97
Renal	49 (52/107)	45 (98/216)	0.59
Neurological	32 (27/85)	31 (55/180)	0.83
Liver	20 (15/76)	18 (30/163)	0.80
Coagulation	15 (16/106)	17 (37/212)	0.61
**Delta SOFA ^h^**, n (%)	46 (43)	83 (39)	0.64
**Type of Infection**, n (%)			
Ventilator-associated pneumonia	51 (47)	22 (10)	0.001 *
Vascular catheter infection	18 (17)	31 (14)	0.52
Surgical infection	17 (16)	42 (19)	0.47
Non-ventilator hospital-acquired pneumonia	14 (13)	51 (23)	0.04 *
Tracheobronchitis	14 (13)	14 (6)	0.05
Catheter-associated UTI	6 (6)	6 (3)	0.20
Sinusitis	6 (6)	5 (2)	0.14
Soft tissue infection	3 (3)	10 (5)	0.47
Intra-abdominal infection	3 (3)	7 (3)	0.85
Endocarditis	2 (2)	2 (1)	0.50
Mastoiditis	2 (2)	5 (2)	0.85
Non-catheter associated UTI	1 (1)	9 (4)	0.12
Osteomyelitis	1 (1)	4 (2)	0.54
Undetermined focus	0 (0)	43 (20)	-
Others	0 (0)	4 (2) ^j^	-
**Concurrent infection**, n (%)			
Bacterial	21 (20)	24 (11)	0.03 *
Viral	3 (3)	9 (4)	0.55
Fungal	7 (7)	6 (3)	0.10
**Serum PCR ^g^** (mg/dL), median (range)	4.8 (1–31)	4 (1–31)	0.25

^a^ Comorbidities or conditions diagnosed prior to the initial blood culture collection during the hospital stay; ^b^ nosocomial diarrhea (three or more daily stool episodes for two or more days); ^c^ >10 mg prednisone for more than 50 days, >40 mg corticosteroid for more than 7 days, or immunomodulatory agent (examples: monoclonal agents, methotrexate); ^d^ granulocytes < 500 cells/mm^3^; ^e^ prior to the initial blood culture collection; ^f^ within 24 h of the initial blood culture collection (at least two of the following criteria: body temperature > 38 °C or <36 °C, heart rate > 90 beats/minute, breath rate > 20 breaths/min or PaCO_2_ < 32 mmHg, white blood cell count > 12,000 cells/mm^3^ or <4000 cells/mm^3^, or ≥10% immature neutrophils); ^g^ within 24 h of the initial blood culture collection; ^h^ in relation to the SOFA at ICU admission; ^i^ Pearson’s chi-square test, Fisher’s exact test, or Mann–Whitney–Wilcoxon U test was used, as required and considering *p* < 0.05 as statistically significant (in bold). Abbreviations: AIDS, acquired immunodeficiency syndrome; CR-GNB, carbapenem-resistant Gram-negative bacilli; PCR, C-reactive protein; SIRS, Systemic Inflammatory Response Syndrome; SOFA, Sequential Organ Failure Assessment. ^j^ Pleural empyema (n = 2), vascular fistula (n = 1), and otitis (n = 1). * in bold statistically significant test.

**Table 3:** Accuracy of the predictive score for the identification of CR-GNB sepsis, stratified by cutoff values of the score.

Cutoff	Sensibility % (95% CI)	Specificity % (95% CI)	False Negative (n)	False Positive (n)	NPV % (95% CI)	PPV % (95% CI)	Youden’s Index ^6^	Rate of Accuracy % (95% CI)
≥1	100 (97–100)	15 (11–20)	0	188	100 (90–100)	37 (31–42)	0.149	43 (38–48)
≥2	99 (95–100)	31 (26–37)	1	136	99 (94–100)	36 (31–42)	0.302	50 (45–55)
≥3	92 (85–96)	65 (58–71)	9	78	94 (89–97)	56 (49–63)	0.564	74 (69–78)
4	66 (56–74)	76 (70–81)	37	53	82 (76–87)	57 (49–66)	0.418	73 (68–77)

Abbreviations: NPV, negative predictive value; PPV, positive predictive value.

## Data Availability

The datasets used and/or analyzed during the current study are available from the corresponding author on reasonable request.

## Supplementary Materials

The following are available online at https://www.mdpi.com/article/10.3390/antibiotics12010021/s1: Figure S1. Distribution of infectious focus in episodes of sepsis caused by CR-GNB (n = 137 infectious focus) and by another or undetermined etiology (n = 212 infectious focus); Figure S2. Plausibility of infectious focus in CR-GNB and non-CR-GNB sepsis, according to the adapted criteria of Klein Klouwenberg et al., (2013); Figure S3. Etiological agents in episodes of CR-GNB sepsis (in orange and blue: n = 162 isolates in 108 episodes) and non-CR-GNB sepsis (in gray: n = 120 isolates in 97 episodes); Figure S4. Polymyxin and carbapenem usage before (3 days preceding) and after (empirical therapy) initial blood culture in CR-GNB sepsis (n = 108 episodes) and non-CR-GNB sepsis (n = 217 episodes); Figure S5. Polymyxin and carbapenem usage before (3 days preceding) and after (empirical therapy) initial blood culture, according to the etiology of sepsis; Figure S6. Polymyxin and carbapenem usage before (3 days preceding) and after (empirical therapy) initial blood culture, according to predictive score; Figure S7. Adequate empirical antimicrobial therapy according to the etiology of sepsis and the predictive score for CR-GNB sepsis; Table S1. Sepsis-2 and adapted sepsis-3 criteria; Table S2. Score for assessing the risk of CR-GNB sepsis, according to the logistic regression coefficients; Table S3. Performance of the clinical–epidemiological score ≥ 3 to predict CR-GNB sepsis, according with the etiology of sepsis, score distribution, and prior use of polymyxins; Table S4. Polymyxins, carbapenems, and adequate empirical antimicrobial therapy according to the performance of the score ≥ 3 in predicting CR-GNB sepsis; Table S5. Pre-test and post-test adequate empirical polymyxin therapy, according to the score ≥ 3 in predicting CR-GNB sepsis. References [5,13,15,16,39,40] are cited in the Supplementary Materials.

## Abbreviations

AUC: area under the curve; BSI: bloodstream infection; CI: confidence interval; CS-GNB: carbapenem-susceptible Gram-negative bacteria; CR: carbapenem-resistant Gram-negative bacteria; CRE: carbapenem-resistant *Enterobacteriaceae*; EAT: empirical antimicrobial therapy; GNB: Gram-negative bacilli; ICU: intensive care unit; IOC/FIOCRUZ: Instituto Oswaldo Cruz, Fundação Oswaldo Cruz; NPV: negative predictive value; NNT: number needed to treat; PPV: positive predictive value; ROC: receiver operating characteristic curve; STROBE: Strengthening the Reporting of Observational Studies in Epidemiology; SAPS-3: Simplified Acute Physiology Score III; SIRS: Systemic Inflammatory Response Syndrome; SSI: surgical site infection; SOFA: sequential organ failure assessment; qSOFA: quick SOFA; UTI: urinary-tract infection; VAP: ventilator-associated pneumonia.
