# Supplementary material for: Predictive Score for Carbapenem-Resistant Gram-Negative Bacilli Sepsis: Single-Center Prospective Cohort Study
Source: Antibiotics (Basel). 2022 Dec 23;12(1):21. doi: 10.3390/antibiotics12010021 (PMC9854893; doi:10.3390/antibiotics12010021)
Supplement: Supplementary file 1 [file antibiotics-12-00021-s001.zip › antibiotics-1890249-supplementary.pdf]

Supplementary File

Table S1. Criteria of sepsis-2[1,2] and sepsis-3 adapted from Singer *et al*. 2016[3]

	**Clinical and laboratorial parameters**
SEPIS-2 (prospectively applied)	**General parameters**: Fever (temperature > 38°C) or hypothermia (temperature <36°C); heart rate > 90 bpm; respiratory rate > 20 bpm; altered mental status (GCS score < 15 points); hyperglycemia (plasma glucose > 140 mg/dl) without diabetes; significant edema or positive fluid balance (>20 ml/kg in 24 h)<br>**Inflammatory parameters**: leukocytes >12,000 cel/ μ L or < 4,000 cel/ μ L or ≥ 10% immature (band) forms; plasma C-reactive protein >2 SD above the normal value; plasma procalcitonin >2 SD above normal value (not available during the study)<br><br>**Hemodynamic parameters**: Arterial hypotension (SBP < 90 mmHg, MAP < 70 mmHg, or a SBP decrease > 40 mmHg in adults or < 2 SD below normal for age)<br>**Organ dysfunction parameters**: Arterial hypoxemia (PaO2/FIO2 < 300 mmHg); acute oliguria (urine output < 0.5 mL/kg/h for at least 2 h despite adequate fluid resuscitation); creatinine increase > 0.5 mg/dL; coagulation abnormalities (INR > 1.5 or aPTT > 60s); Ileus (absent bowel sounds) thrombocytopenia (platelets count < 100,000/μL); hyperbilirubinemia (plasma total bilirubin >4 mg/dl)<br>**Tissue perfusion parameters**: Hyperlactatemia (arterial lactate >1 mmol/l); decrease capillary refill or mottling<br>**Sepsis**<br>Infection suspected or documented and two or more of the general or inflammatory parameters<br>**Severe Sepsis**<br>Sepsis with one or more organ dysfunction parameters<br>**Septic Shock**<br>Sepsis with persistent arterial hypotension that requires inotropes or vasopressor, with or without serum lactate level greater than 2mmol/L, despite adequate fluid resuscitation
**Adapted SEPSIS-3** (retrospectively applied)	**QuickSOFA**<br>(applied to patients without **mechani**cal ventilation and/or sedation, within 72 hours before and 24 hours after the date of blood culture)<br>**Presence ≥ 2 of the following variables:**<br>SBP < 100mmHg; Respiratory rate > 22 irpm; GCS score < 15<br><br>Delta SOFA points ≥ 2<br>between SOFA scores measured on two calendar days, between the period of 72 hours that preceded to 24 hours that succeeded the date of initial blood culture, and the ICU admission date

Abbreviations: aPTT: activated partial thromboplastin time; GCS: Glasgow Coma Scale; ICU: Intensive Care Unit; INR: international normalized ratio; MAP, mean arterial pressure; SBP: systolic blood pressure; SD, standard deviation; SOFA: Sequential Organ Failure Assessment.

Table S2. Score for assessing the risk of CR-GNB sepsis, according to the logistic regression coefficients[4].

Variables	Regression coefficient (β)	Points
Previous infection,	1.454	1
Mechanical ventilation	1.438	1
Use of carbapenem	1.228	1
Length of hospital stay[a]	0.028	1

[a]19 days as the cut-off of hospitalization time prior to the episode of sepsis that best predicts CR-GNB sepsis (sensitivity and specificity of 73% and 80%, respectively)[5]. CR-GNB, carbapenem-resistant Gram-negative bacilli.

Table S3. Performance of the clinical-epidemiological score ≥ 3 to predict CR-GNB sepsis, according with the etiology of sepsis, score distribution and prior use of polymyxins.

Score ≥ 3	# of sepsis episodes	Etiologic agents of sepsis (# of episodes)	Polymicrobial sepsis, % (#)	Score distribution, # of episodes					Previous polymixins use, % (n)	Polymixins use, 3 days preceding blood culture, % (n)
				**0**	**1**	**2**	**3**	**4**		
True negative or concordant result	143	57% (81/143) undetermined aetiology;<br>33 CS-Enterobacteriaceae (32);<br>24 Gram-positive cocci (23);<br>11 CS-non-fermentative bacteria (11);<br>4 fungi (4);<br>1 *Mycobacterium tuberculosis*;<br>1 Gram-negative cocci;<br>1 Gram-negative coccobacilli	8 (12)	33	52	58	-	-	6 (9/142)	10 (15/142)
False negative	9	6 CR-non-fermentative bacteria (5);<br>4 CR-Enterobacteriaceae species (4);<br>• CR-*Klebsiella pneumoniae* (2);<br>• CR-*K. pneumoniae* and CS-*Providencia stuartii* (1);<br>• CR-*K. pneumoniae,* CS-*E. coli* and CS-*E. cloacae* (1);<br>• CR-*Pseudomonas aeruginosa* (1);<br>• CR-*P. aeruginosa* and *Enterococcus faecalis* (1);<br>• CS-*P. aeruginosa* and *S. maltophilia* (1);<br>• CR-*Acinetobacter baumannii* (2)	44 (4)	-	1	8	-	-	0 (0)	22 (2)
True positive	99	83 CR-non-fermentative bacteria (71);<br>33 CR-Enterobacteriaceae species (31)	42 (42)	-	-	-	28	71	63 (62)	41 (41)
False positive or discordant result	78	54% (42/78) undetermined aetiology;<br>19 Gram-positive cocci (13);<br>12 non-fermentative bacteria (12);<br>6 fungi (5);<br>5 CS-Enterobacteriaceae species (5);<br>2 *M. tuberculosis* (2);<br>1 anaerobic Gram-positive bacilli	12 (9)	-	-	-	25	53	54 (42)	40 (31)

CR-GNB, carbapenem-resistant Gram-negative bacilli; CS, carbapenem susceptible

Table S4. Polymyxins, carbapenems and adequate empirical antimicrobial Table S3. in predicting CR-GNB sepsis.

Score ≥ 3	# of sepsis episodes	Score distribution, # of episodes					Empirical polymyxins therapy, n (%)	Empirical carbapenems therapy, n (%)	Adequate EAT, % (n)
		0	1	2	3	4			
Concordant result	143	33	52	58	-	-	55 (38)	105 (73)	-
True negative[a]	62	13	19	30	-	-	32 (52)	50 (81)	55 (89)
False negative	9	-	1	8	-	-	6 (67)	7 (78)	8 (89)
True positive	99	-	-	-	28	71	79 (80)	76 (77)	73 (74)
Discordant result	78	-	-	-	25	53	53 (68)	59 (76)	-
False positive[a]	35	-	-	-	4	31	26 (74)	23 (66)	22 (63)

[a]excluding sepsis with undetermined etiology. CR-GNB, carbapenem-resistant Gram-negative bacilli

Table S5. Pre-test and post-test adequate empirical polymyxins therapy, according to the score ≥ 3 in predicting CR-GNB sepsis.

Score ≥ 3	# of sepsis episodes, n (%)	Score distribution, # of episodes					Empirical polymyxins therapy, n (%)	Pre-test adequate empiric polymyxins, n (%)	Post-test adequate empiric polymyxins, n (%)	Score adequacy rate improvement, n (%)[b]
		0	1	2	3	4				
True negative[a]	62 (30)	13	19	30	-	-	32 (52)	0	32 (withdraw therapy)	32 (52%)
False negative	9 (4)	-	1	8	-	-	6 (67)	6	0	0
True positive	99 (48)	-	-	-	28	71	79 (80)	79	99	20 (20%)
False positive[a]	35 (17)	-	-	-	4	31	26 (74)	0	0	0
Total, # (%)	205 (100)							85 (42)	131 (64)	46 (22%)

[a]excluding sepsis with undetermined etiology. CR-GNB, carbapenem-resistant Gram-negative bacilli; [b]compared to clinical practice (post-test minus pre-test result)

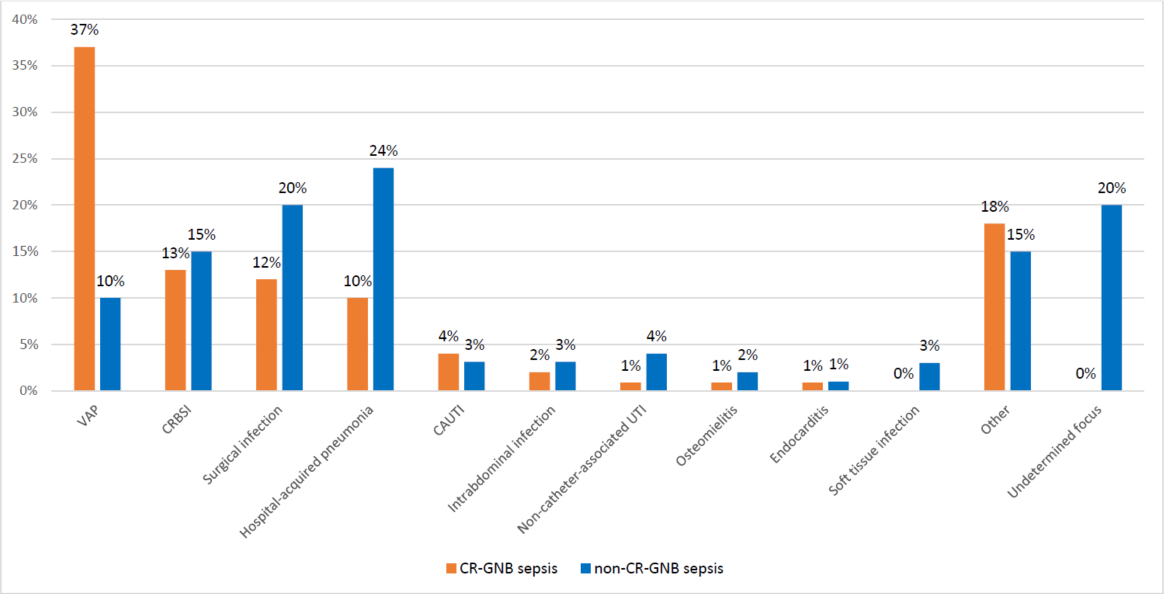

Figure S1. Distribution of infectious focus in episodes of sepsis caused by CR-GNB (n=137 infectious focus) and by another or undetermined etiology (n=212 infectious focus). CAUTI, catheter-associated urinary tract infection; CRBSI, catheter-related bloodstream infection; CR-GNB, carbapenem-resistant Gram-negative bacilli; UTI, urinary tract infection; VAP, ventilator-associated pneumonia.

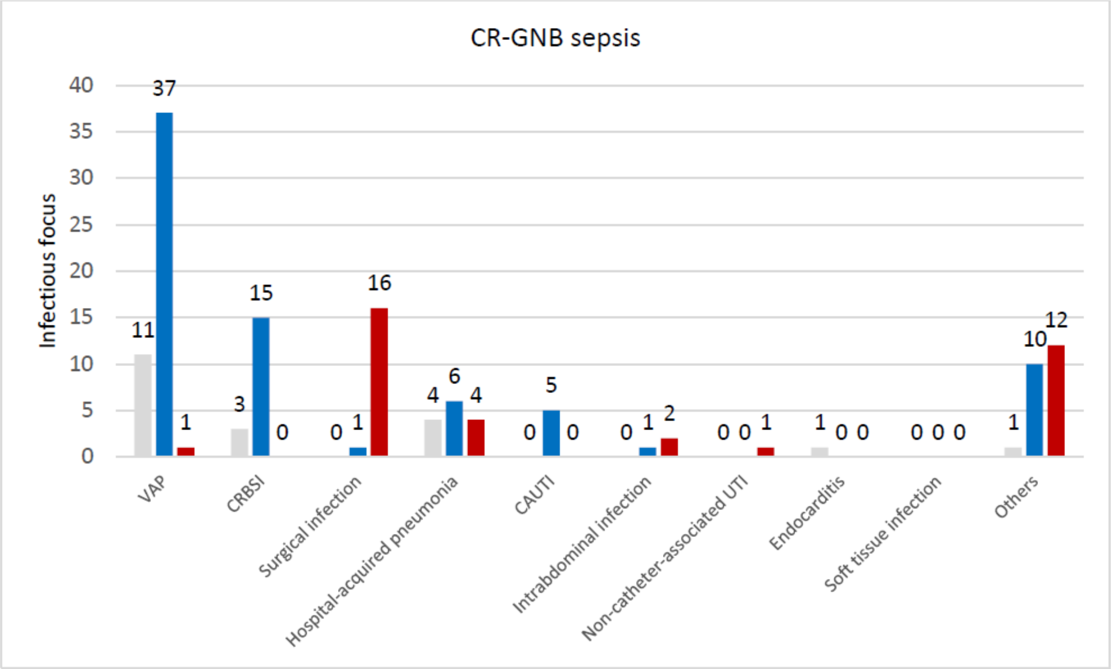

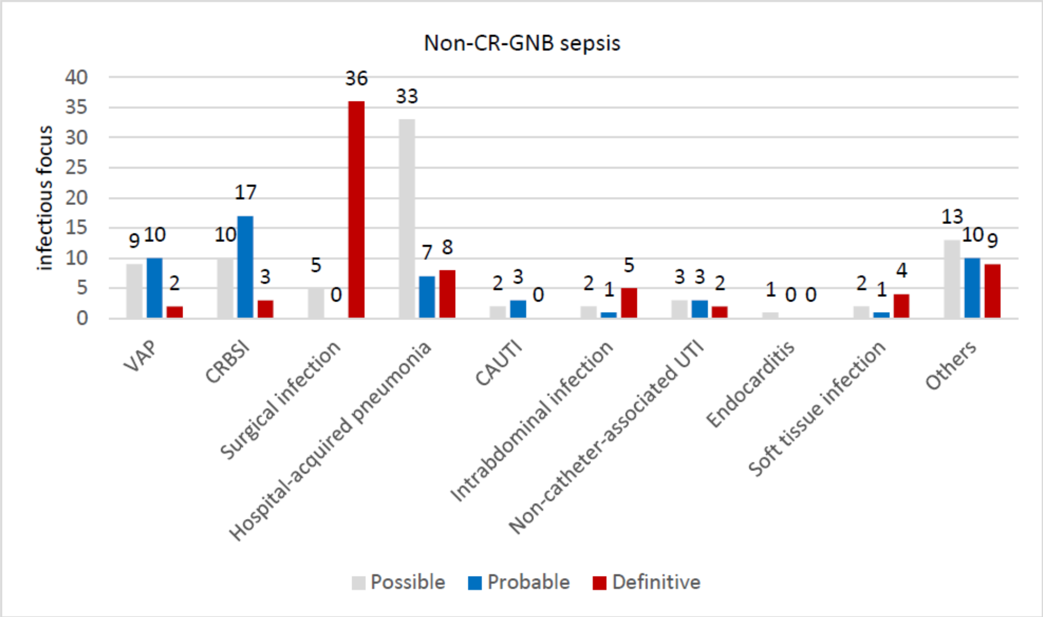

Figure S2. Plausibility of infectious focus in CR-GNB and non-CR-GNB sepsis, according to the adapted criteria of Klein Klouwenberg *et al.* (2013)[6]. CAUTI, catheter-associated urinary tract infection; CS-GNB, carbapenem-susceptible Gram-negative bacilli; CRBSI, catheter-related bloodstream infection; CR-GNB, carbapenem-resistant Gram-negative bacilli; UTI, urinary tract infection; VAP, ventilator-associated pneumonia.

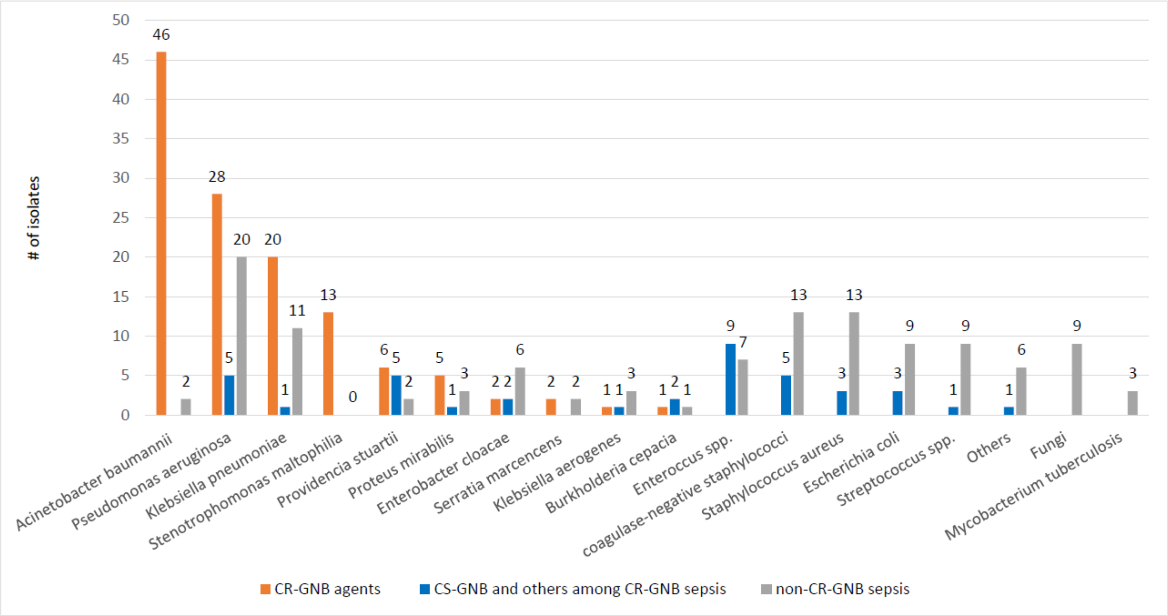

Figure S3. Etiological agents in episodes of CR-GNB sepsis (in orange and blue: n=162 isolates in 108 episodes) and non-CR-GNB sepsis (in gray: n=119 isolates in 97 episodes). Others in CR-GNB sepsis: Gram-positive cocci (n=1). Others in non-CR-GNB sepsis: *Citrobacter koseri*, *Klebsiella oxitoca*, *Staphylococcus sp.*, *Moraxella sp.*, *Haemophilus sp.* and anaerobic Gram-positive bacilli. CR-GNB, carbapenem-resistant Gram-negative bacilli; CS-GNB, carbapenem susceptible Gram-negative bacilli.

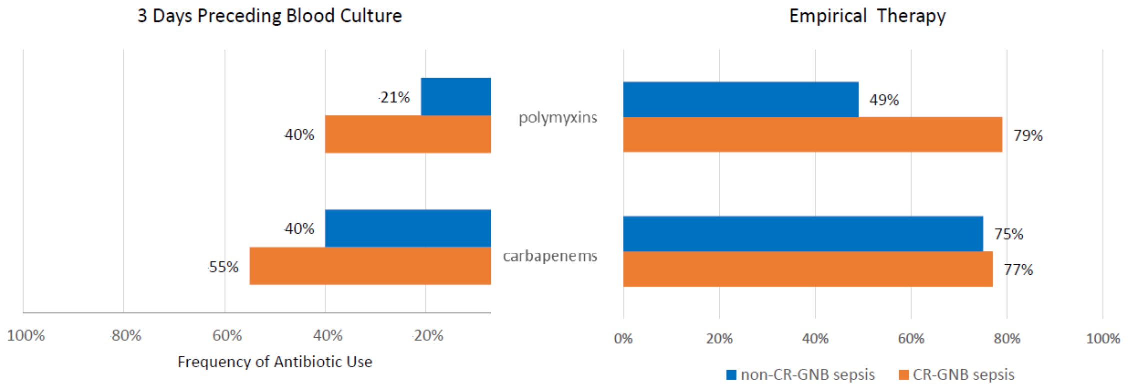

Figure S4. Polymyxins and carbapenems usage before (3 days preceding) and after (empirical therapy) initial blood culture in CR-GNB sepsis (n=108 episodes) and non-CR-GNB sepsis (n=217 episodes). CR-GNB, carbapenem-resistant Gram-negative bacilli.

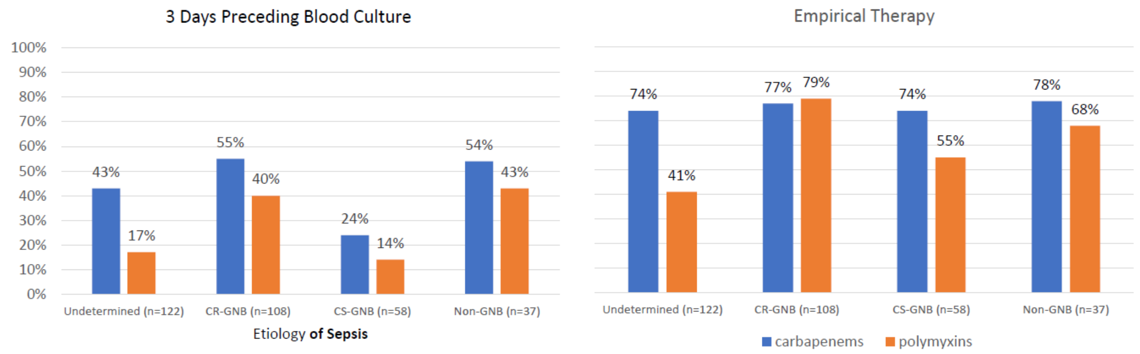

Figure S5. Polymyxins and carbapenems usage before (3 days preceding) and after (empirical therapy) initial blood culture, according to the etiology of sepsis. CR-GNB, carbapenem-resistant Gram-negative bacilli, CS-GNB, carbapenem-susceptible Gram-negative bacilli.

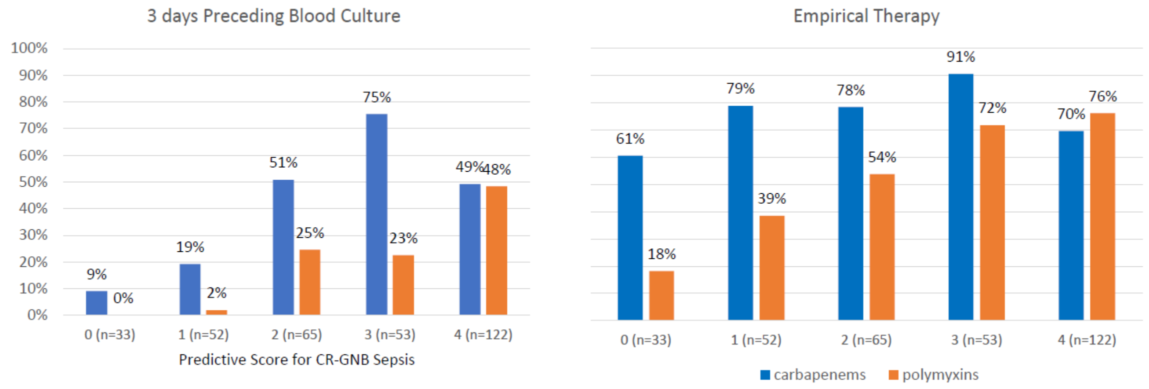

Figure S6. Polymyxins and carbapenems usage before (3 days preceding) and after (empirical therapy) initial blood culture, according to predictive score. CR-GNB, carbapenem-resistant Gram-negative bacilli.

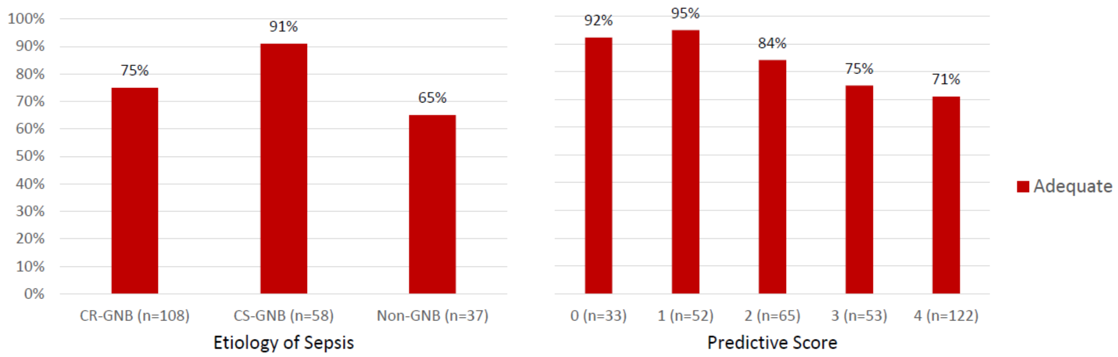

Figure S7. Adequate empirical antimicrobial therapy according to the etiology of sepsis and the predictive score for CR-GNB sepsis. CR-GNB, carbapenem-resistant Gram-negative bacilli.

References

1. 1 Monti G LG, Taddeo D, Isella F, Zangrillo A. Clinical Aspects of Sepsis: An Overview. In *Sepsis Diagnositic Methods and Protocols* (ed. Walker J) London: Humana Press - Springer Science, 2015. p.17-33.
2. 2 Levy, M. M. *et al.* 2001 SCCM/ESICM/ACCP/ATS/SIS International Sepsis Definitions Conference. *Critical care medicine* **31**, 1250-1256, doi:10.1097/01.CCM.0000050454.01978.3B (2003).
3. 3 Singer, M. *et al.* The Third International Consensus Definitions for Sepsis and Septic Shock (Sepsis-3). *JAMA* **315**, 801-810, doi:10.1001/jama.2016.0287 (2016).
4. 4 Lima, E. M. *et al.* Predictive factors for sepsis by carbapenem resistant Gram-negative bacilli in adult critical patients in Rio de Janeiro: a case-case-control design in a prospective cohort study. *Antimicrob Resist Infect Control* **9**, 132, doi:10.1186/s13756-020-00791-w (2020).
5. 5 Lima, E. M. Fatores Preditivos para Sepse por Bacilos Gram-Negativos Resistentes aos Carbapenemas em Pacientes Adultos Críticos do Rio de Janeiro Master Science, Instituto Oswaldo Cruz, Fundação Oswaldo Cruz, Rio de Janeiro, Brazil (2017). Microsoft Word - Dissertação mestrado versão jun 2018.docx (fiocruz.br). Accessed 12 August 2022.
6. 6 Klein Klouwenberg, P. M. *et al.* Interobserver agreement of Centers for Disease Control and Prevention criteria for classifying infections in critically ill patients. *Critical Care Medicine* **41**, 2373-2378, doi:10.1097/CCM.0b013e3182923712 (2013).
